# Supplementary material for: Differential regulation of coronal and lambdoid suture patency by PTHLH and HHIP activity in mice
Source: Development. 2025 Oct 13;152(19):dev204875. doi: 10.1242/dev.204875 (PMC12579929; doi:10.1242/dev.204875)
Supplement: Supplementary information [file develop-152-204875-s1.pdf]

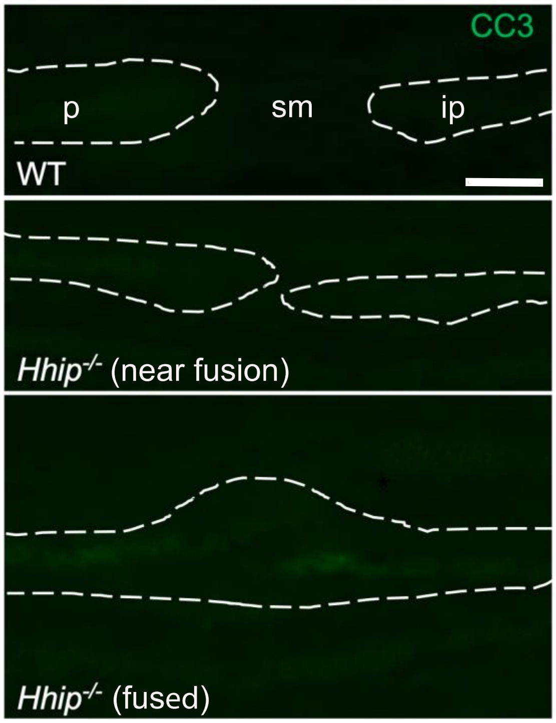

Fig. S1. Lack of apoptosis in the *Hhip*$^{-/-}$ lambdoid suture. Cleaved caspase 3 IHC (CC3, green) for apoptotic cells of WT (top), *Hhip*$^{-/-}$ near fusion (middle) and fused (bottom) lambdoid sutures. n=3 WT and 3 *Hhip*$^{-/-}$. White dashed lines indicate ip, interparietal, and p, parietal bones. sm, suture mesenchyme. Sections are in the transverse plane. Scale bar: 50 μm.

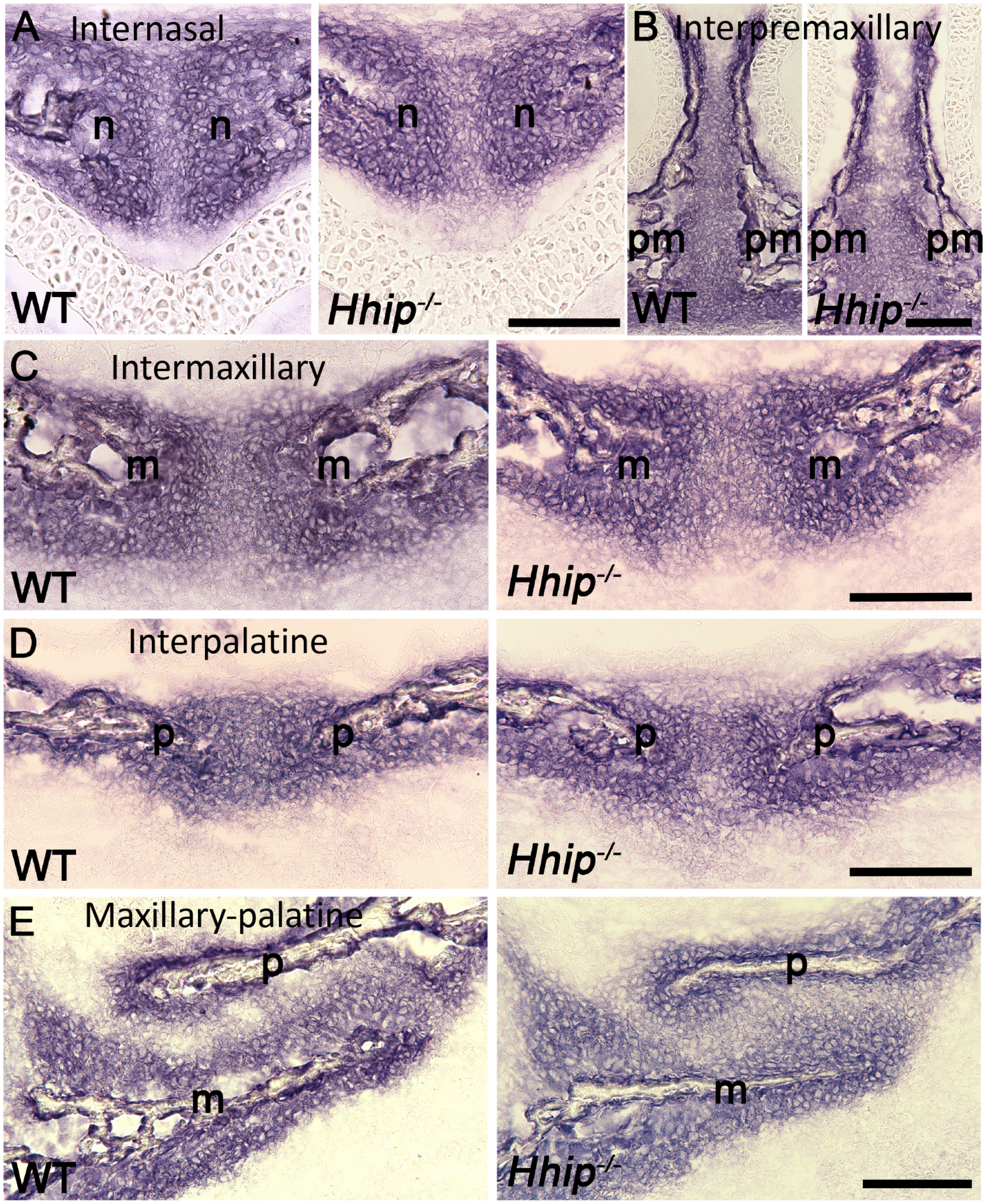

Fig. S2. *Hhip*$^{-/-}$ facial sutures are unfused at E18.5. ALP activity (blue) of preosteoblasts and osteoblasts in (A) Internasal, (B) Interpremaxillary, (C) Intermaxillary, (D) Interpalatine, and (E) Maxillary-palatine sutures. n=3 WT and 3 *Hhip*$^{-/-}$. Bone abbreviations: n, nasal; pm, premaxillary; m, maxillary; p, palatine. Sections are in the coronal plane. Scale bars: 100 μm.

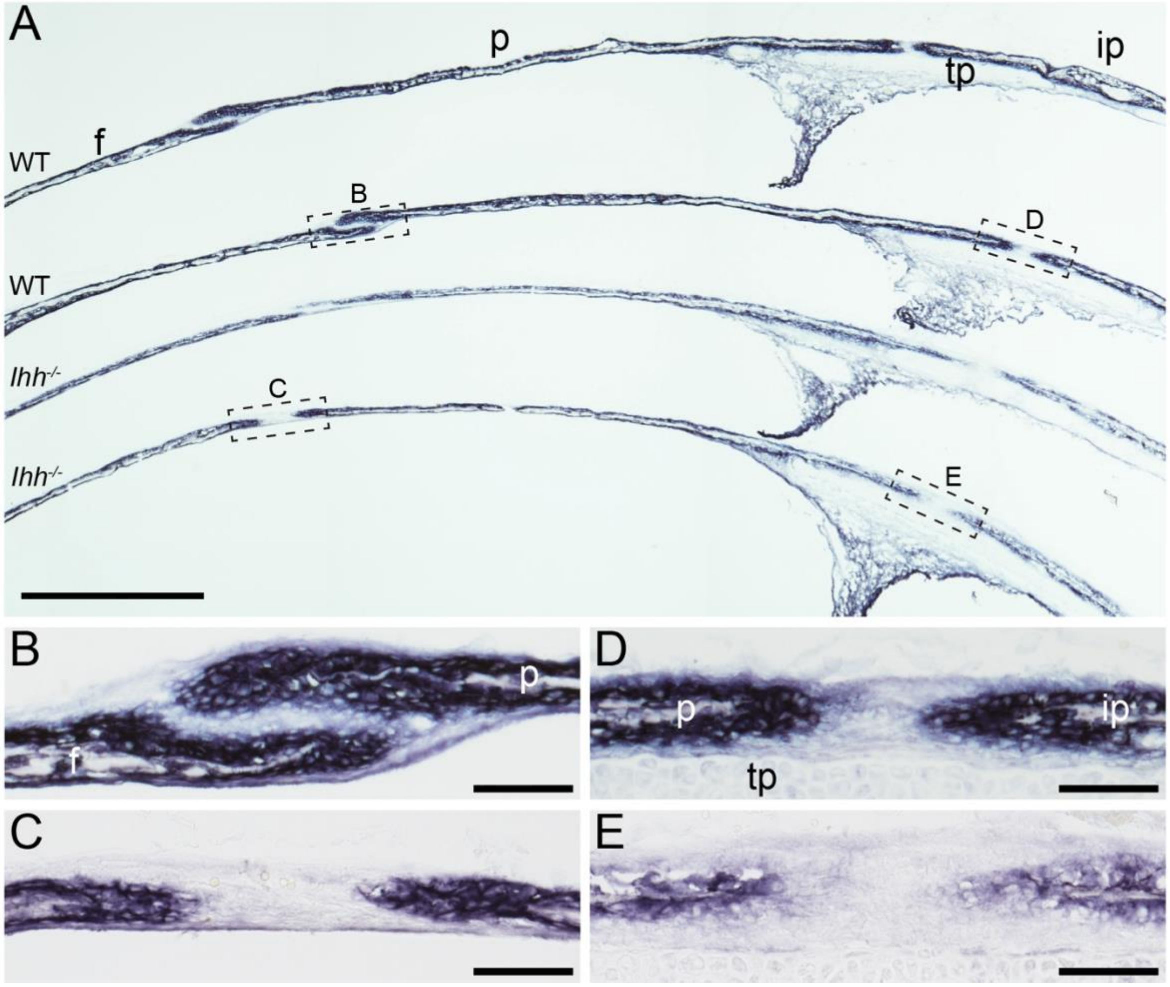

Fig. S3. Loss of *Ihh* impairs E18.5 coronal and lambdoid suture development. (A) Representative examples of WT (n=5) and *Ihh*$^{-/-}$ (n=7) calvariae stained for ALP activity (blue). Dashed rectangles indicate regions enlarged in B-E. Panel is a composite of multiple images. (B) WT and (C) *Ihh*$^{-/-}$ coronal sutures. (D) WT and (E) *Ihh*$^{-/-}$ lambdoid sutures. f, frontal bone; ip, interparietal bone; p, parietal bone; tp, tectum posterius. Sections are in the sagittal plane. Scale bars: A, 500 µm; B-E, 50 µm.

Development • Supplementary information

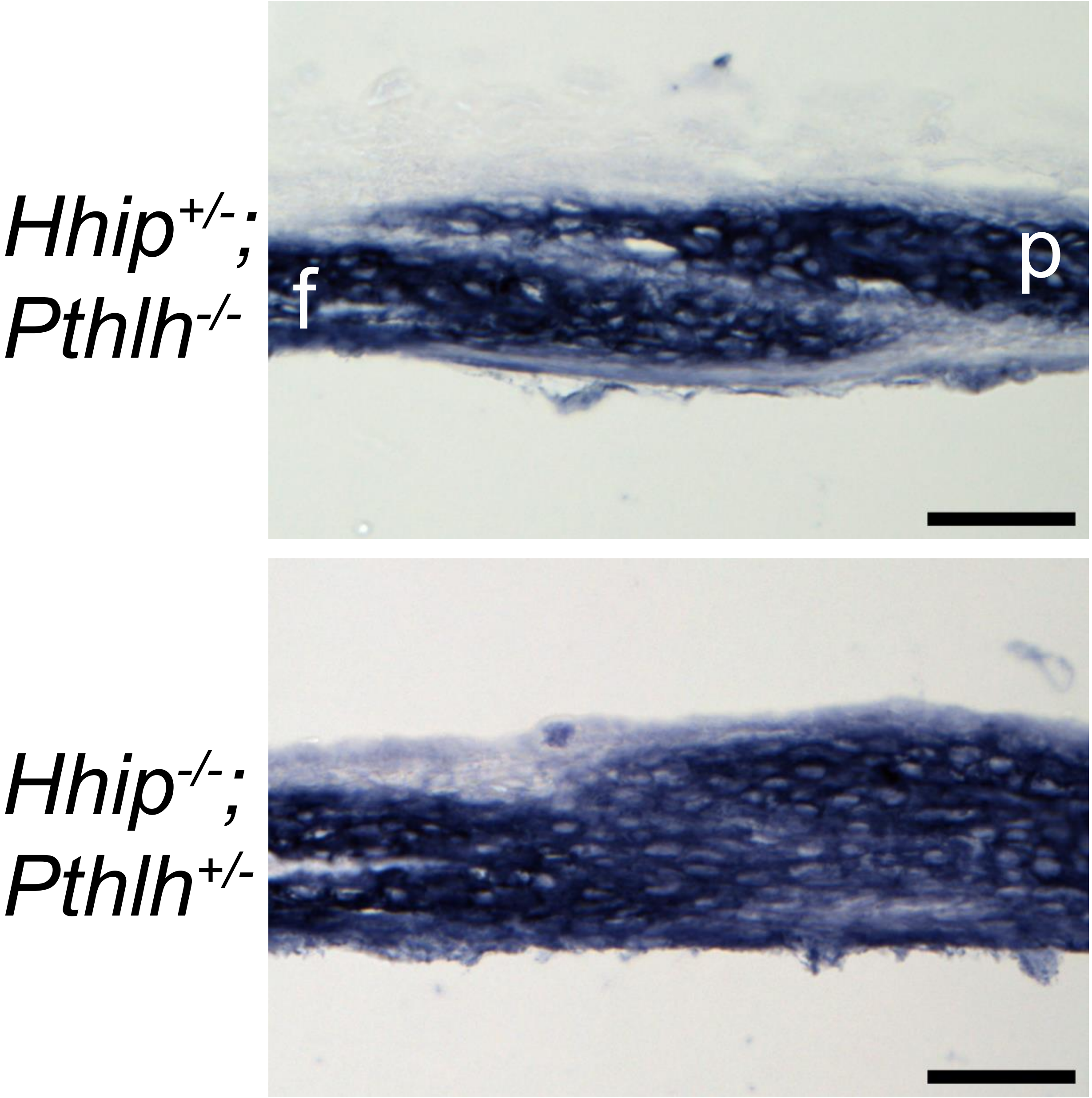

Fig. S4. Coronal suture fusion requires full deletion of both *Pthlh* and *Hhip*. ALP activity (blue) in *Hhip*$^{+/-}$;*Pthlh*$^{-/-}$ (n=3) and *Hhip*$^{-/-}$;*Pthlh*$^{+/-}$ (n=1) E18.5 coronal sutures. Abbreviations: f, frontal bone; p, parietal bone. Sections are in the sagittal plane. Scale bars: 50 µm.

Development • Supplementary information

Table S1. Anatomical definitions of skull landmarks used for PCA.

Bilateral Landmarks	Anatomical Definition
lnasapl, rnasapl	Most supero-anterior point of the premaxilla accounting for the lateral part of the nasal aperture
lfppm, rfppm	Most supero-posterior point of the premaxilla accounting for the lateral part of the nasal aperture
lnslp, rnslp	Most postero-medial point of the nasal bone
lnsla, rnsla	Most antero-medial point of the left nasal bone
liohd, riohd	Most distal point of the infraorbital hiatus
lflac, rflac	Intersection of frontal process of maxilla with frontal and lacrimal bones, taken on the maxilla
lzyt, rzyt	Intersection of zygoma with zygomatic process of temporal, taken on zygoma
lpfl, rpfl	Most lateral intersection of the frontal and parietal bones, taken on the parietal
lsqu, rsqu	Most superior point on the squamous temporal, intersection of the coronal suture
lpsq, rpsq	Most posterior point on the posterior extension of the forming squamosal
lpfm, rpfm	Most medial intersection of the frontal and parietal bones, taken on the parietal
lpto, rpto	Most postero-medial point on the parietal
loci, roci	The superior posterior point on the ectocranial surface of the occipital lateralis on the foramen magnum
lva, rva	Most posterior point on the ala of the vomer
lalf, ralf	Most anteromedial point on the frontal bone
lasph, rasph	Postero-medial point of the inferior portion of the alisphenoid
lsyn, rsyn	Most antero-lateral point on the corner of the basioccipital

Individual Landmarks	Anatomical Definition
ethma	Anterior most point on the body of the vomer, taken on the ventral surface
intpar	Most anterior point on the ectocranial surface of the interparietal on the midsagittal plane
ans	Anterior nasal spine is the most anterior point of interpremaxillary suture at base of nasal aperture, midline
amsph	Most antero-medial point on the body of the sphenoid
bas	Mid-point on the anterior margin of the foramen magnum, taken on basioccipital

Development • Supplementary information

Table S2. Suture Fusion Frequency.

Available for download at
https://journals.biologists.com/dev/article-lookup/doi/10.1242/dev.204875#supplementary-data

Table S3. Differential gene expression and gene ontology enrichments for bulk RNA-seq analysis of the coronal and lambdoid *Hhip*$^{-/-}$ suture.

Available for download at
https://journals.biologists.com/dev/article-lookup/doi/10.1242/dev.204875#supplementary-data

Table S4. Common and unique differential gene expression and gene ontology enrichments between the coronal and lambdoid *Hhip*$^{-/-}$ sutures (relative to WT).

Available for download at
https://journals.biologists.com/dev/article-lookup/doi/10.1242/dev.204875#supplementary-data
